# Adaptive and self-learning Bayesian filtering algorithm to statistically characterize and improve signal-to-noise ratio of heart-rate data in wearable devices

**DOI:** 10.1098/rsif.2024.0222

**Published:** 2024-09-04

**Authors:** Luca Cossu, Giacomo Cappon, Andrea Facchinetti

**Affiliations:** ^1^ Department of Information Engineering, University of Padova, Padova, Italy

**Keywords:** Bayesian filtering, heart-rate data, signal processing, noise reduction, autoregressive model

## Abstract

The use of wearable sensors to monitor vital signs is increasingly important in assessing individual health. However, their accuracy often falls short of that of dedicated medical devices, limiting their usefulness in a clinical setting. This study introduces a new Bayesian filtering (BF) algorithm that is designed to learn the statistical characteristics of signal and noise, allowing for optimal smoothing. The algorithm is able to adapt to changes in the signal-to-noise ratio (SNR) over time, improving performance through windowed analysis and Bayesian criterion-based smoothing. By evaluating the algorithm on heart-rate (HR) data collected from Garmin Vivoactive 4 smartwatches worn by individuals with amyotrophic lateral sclerosis and multiple sclerosis, it is demonstrated that BF provides superior SNR tracking and smoothing compared with non-adaptive methods. The results show that BF accurately captures SNR variability, reducing the root mean square error from 2.84 bpm to 1.21 bpm and the mean absolute relative error from 3.46% to 1.36%. These findings highlight the potential of BF as a preprocessing tool to enhance signal quality from wearable sensors, particularly in HR data, thereby expanding their applications in clinical and research settings.

## 1. Introduction

Wearable devices have witnessed a surge in popularity in the last few years, thanks to their capacity to provide continuous tracking of vital signs and to obtain insights into users’ health. Indeed, wearable devices allow monitoring a broad spectrum of health parameters, encompassing metabolic, cardiovascular and gastrointestinal health, as well as aspects related to sleep, neurology and exposure to environmental factors [1,2]. As a result, wearable devices have emerged as prevalent choices for health tracking [3].

The versatility of these devices extends beyond personal use. They hold the potential for integration into medical surveillance, facilitating noninvasive medical care and enabling mobile health and wellness monitoring [4]. Furthermore, wearable devices are increasingly being explored for their potential in remote patient monitoring, a capability that can lead to earlier diagnosis and proactive/personalized interventions [5,6].

Analysis of heart rate (HR) is well known to be fundamental for assessing the overall cardiovascular health of a patient, and, through wearable devices, raw HR data can be easily obtained and processed to extract meaningful information and insights [7]. In this scenario, accurate signal processing techniques are necessary to reduce the noise present in raw HR data [8] and effectively extract high-level features to be used, in a second phase, as input to clinical decision support systems able to interpret patients’ health status and ultimately provide personalized interventions [9].

One commonly used technique for health-related signal processing is the moving average method, which involves calculating the average of a certain number of samples over a predefined time window [10–12]. However, this method has limitations, such as the inability to capture variations in time of the signal-to-noise (SNR) ratio and the fact of potentially being biased by the presence of very pronounced peaks/nadirs in the data. Another possibility is adapting Bayesian filtering (BF) approaches. BF is a signal processing technique that has shown to be effective in reducing noise present in biosignals, such as myoelecric signals [13] and continuous glucose monitoring sensors [14,15], surpassing the limitations of moving average-based approaches.

In this study, we present a novel adaptive and self-learning BF technique to smooth HR data gathered from wearable devices and estimate the statistical characteristics of both the signal and noise components.

## 2. Material and methods

In the following section, we will provide a comprehensive overview of both the new BF algorithm and the procedure developed to assess its performance. We will start by describing how we generated an *in silico* dataset of HR signals that allows quantifying the performance of BF algorithm versus a ground truth signal and, then, we will move to the description of the BF technique, highlighting its principal features which are being adaptive, i.e. the ability to track variations of the SNR over time, and being self-learning, i.e. the estimation of the SNR is performed automatically thanks to a Bayesian criterion.

### 2.1. The dataset

The HR data utilized in this study were collected with a Garmin Vivoactive 4 (Garmin Ltd., Olathe, KA, USA) smartwatch worn by three patients affected by amyotrophic lateral sclerosis and multiple sclerosis for 7 days, providing a comprehensive and dynamic representation of subjects’ HR dynamics that can be collected via smartwatch in a pathological population. The subjects gave their informed consent for inclusion before they participated in the study. The study was conducted in accordance with the Declaration of Helsinki, and the protocol was approved by the Ethics Committee of A.O.U. Città della salute e della scienza di Torino (314/2021).

The Garmin Vivoactive 4 is a widely used wearable device known for its accurate HR monitoring capabilities and compatibility with various health and fitness applications. The smartwatch recorded HR measurements at a sampling rate of 30 s. The extended duration of the dataset enables the analysis of long-term HR patterns, including diurnal variations, responses to physical activities and potential changes in HR variability across different periods.

The dataset has been split into two parts. The first subset consists of 4 days from the whole dataset, which were carefully chosen to capture a diverse range of HR patterns and activities, including rest periods, exercise and potential physiological responses to stressors. This subset served as basis to create the synthetic HR data, which will be used to quantify the performance of BF algorithm, ensuring its adaptability to various HR dynamics. The second dataset consists of the remaining days from the whole dataset, which will be used to test on real-life HR time-series the adaptive and self-learning abilities of BF algorithm.

### 2.2. Generation of synthetic heart-rate signals

Accurate evaluation and benchmarking of signal processing techniques necessitate the extraction of a reliable ground truth signal that will serve as a target signal to be reconstructed by BF algorithm. Unfortunately, as in many biomedical applications, also in this case the availability of a ground truth HR dataset is not available. However, this limitation can be overcome by resorting to the *in silico* extraction of HR ground truth, from the raw signal itself, with ad hoc filtering procedures. In this paper, to obtain a ground truth HR signal, we decided to adopt the following procedure.

**Figure 1.**
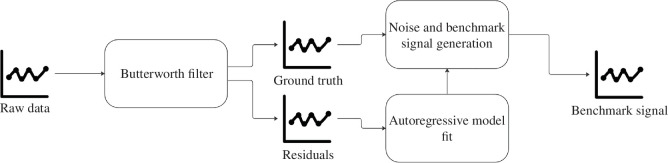
Steps to generate the benchmark signal. Starting from filtering of the raw signal we fit an autoregressive model on the residuals. The estimated noise variance is then used to generate Gaussian noise on the filtered signal to obtain the benchmark signal.

The first step was the application of a sixth-order Butterworth filter with a cutting frequency of 0.4 Hz to the raw HR data (first step of figure 1). The objective of this step was to attenuate high-frequency noise and artifacts while preserving the essential components of the HR signal. The smoothed HR signal obtained in this represents the *in silico* ground truth HR time-series. The selection of the filter order and cutting frequency was based on a trial-and-error procedure, aimed to find a trade-off between noise reduction and the preservation of relevant physiological information. This procedure was supported by visual inspection of the results by experts to ensure that meaningful information of the signal was preserved.

The second step concerned the *in silico* generation of the noise component overlapped to the ground truth HR data. The generation of this noisy component is based on the quantification of the statistical characteristics of HR residuals, i.e. the time-series obtained as difference between the original HR data and the ground truth generated in the first step. To model the autocorrelation of the HR residuals, an autoregressive (AR) model was fitted on the residual traces. In fact, it is well known that AR models are well suited for capturing the temporal dependencies and dynamics inherent in HR data [16], making it a suitable choice for noise estimation in this study. By estimating the parameters of the AR model on the residuals, the underlying noise characteristics were captured and modelled.

In our analysis, we selected an AR order of 20, considering the potential complexity and long-term dependencies present in the residual noise. The selection of the AR order was performed in a data-driven manner, by using a combination of the Akaike information criterion (AIC) [17] and the Anderson test [18] to strike a balance between model complexity and goodness-of-fit, and ensure that the selected AR model adequately captured the statistical properties of the noise residuals.

Of note, the estimated noise variance, derived from the AR model, will be utilized as a guiding factor for adding realistic noise components to the ground truth signal, simulating real-world conditions encountered during HR measurements.

**Figure 2.**
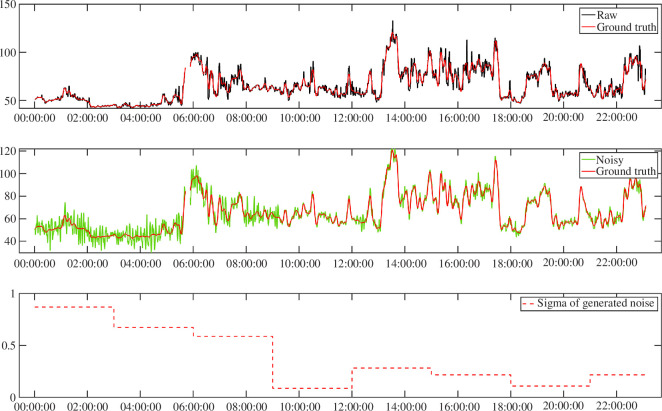
Representative original and *in silico* data. Top panel: original HR data (black) versus ground truth HR signal (red) obtained after the application of step 1 (i.e. after applying the Butterworth filter). Middle panel: ground truth HR signal (red) versus synthetic HR data (green) obtained after the application of one realization of HR noise via AR model. Bottom panel: time varying σ driving the AR model of the added noise.

The resulting benchmarking signal is shown in green in figure 2, while the ground truth HR signal resulting from the Butterworth filter is depicted in black. Incorporating HR noise based on the estimated autocorrelation via AR model allowed for the creation of synthetic HR signals that resembled, from a statistical point of view, the complexities and challenges encountered in practical scenarios. The noise has been generated in a time variant fashion to resemble the possible variations of noise due to different factors of real-life acquisition, such as movement and sweat.

### 2.3. The proposed Bayesian filtering approach for heart-rate data noise reduction

#### 2.3.1. The core: automatic signal-to-noise ratio estimation and smoothing in given time window

BF is an advanced statistical technique used to estimate the hidden or underlying state of a system based on noisy or incomplete observations. It employs the principles of Bayesian inference, which combines prior knowledge about the system’s behaviour with new data to iteratively update and refine the estimated state. In the context of HR data analysis, BF enables the extraction of accurate and robust estimates of HR variations by effectively separating the underlying physiological signal from noise and artifacts. This is achieved by leveraging prior knowledge of HR dynamics, represented by the AR model capturing the temporal dependencies and dynamics of the HR signal [19,20].

We assume that the HR measurements can be represented by a random process *y*(*k*), where y(1),y(2),…,y(k),…,y(n) are the samples collected at regularly spaced time intervals $t=kT_{s}$ , where *k* = 1, 2,…, *n*, and $T_{s}$ is the sampling period for HR. At each sampling time *k*, *y*(*k*) can be expressed as the sum of two components:


(2.1) y(k)=u(k)+w(k).

Here, *u*(*k*) represents the true unknown signal being measured (in this case, the HR) and *w*(*t*) represents the additive measurement noise. Let us define the vectors $y=[y(1),y(2),\ldots ,y(n){]}^{T},u=[u(1),u(2),\ldots ,u(n){]}^{T}$ and $w=[w(1),w(2),\ldots ,w(n){]}^{T}$ . These vectors contain the measured data, the unknown signal and the measurement noise, respectively, collected at time intervals k=1,2,…,n .

In the Bayesian framework, one can estimate *u*(*k*) from the noisy samples *y*(*k*) by utilizing *a priori* statistical information on *u*(*k*) and *w*(*k*) to compute the estimate $\hat{u}$ . This estimate, which is linearly dependent on *y*, minimizes. This estimation, denoted as $\hat{u}$ , is linearly dependent on *y* and aims to minimize $E\;\left[{\left|\left|u-\hat{u}\right|\right|}^{2}\right]$ .

Specifically, if *u* and *w* are uncorrelated zero-mean random vectors with *a priori* covariance matrices denoted by $\Sigma_{u}$ and $\Sigma_{w}$ , respectively, then the linear mean square estimate of *u* given *y* is the solution to the following optimization problem:


(2.2) $$\underset{u}{argmin}{\left(y-u\right)}^{T}\Sigma_{w}^{-1}\left(y-u\right)+u^{T}\Sigma_{u}^{-1}u.$$

This can be simplified to


(2.3) $$\hat{u}={\left(\Sigma_{w}^{-1}+\Sigma_{u}^{-1}\right)}^{-1}\Sigma_{w}^{-1}y.$$

As mentioned earlier, implementing BF begins with defining a statistical prior knowledge on the unknown HR signal and the measurement noise (*u*(*k*) and *w*(*k*) in equation (2.1)). In filtering, smoothing and deconvolution problems, a commonly used *a priori* model to describe a regular physiological signal on a uniformly spaced discrete grid is the multiple-integrated white noise [21,22]. This model is simple yet convenient because the only unknown parameter is the variance of the white noise driving the model, which can be estimated from the data using a smoothing criterion. The number of integrators, cannot be analytically determined. However, in the smoothing problems discussed in the literature, the signal estimate was found to be not very sensitive to this value [21]. Typically, it ranges from 1 to 3, depending on the specific smoothing problem. In our problem, we chose to use a white noise signal cascaded with two integrators, also known as the integrated random walk,


(2.4) u(k)=2u(k−1)−u(k−2)+v(k),

as similarly done in other works applying this technique to other phisiological signals, such as glucose [23,24]. In the equation given above, v(k) represents a zero-mean Gaussian noise with an unknown variance of $\lambda^{2}$ . By using equation (2.4), we can easily calculate $\Sigma_{u}$ :


(2.5) $$\Sigma_{u}=\lambda^{2}{\left(F^{T}F\right)}^{-1}.$$

Here, *F* refers to the square *n*-dimensional lower triangular Toeplitz matrix. The first column of *F* is represented as ${\left[1,-2,1,0,\ldots ,0\right]}^{T}$ .

To describe the measurement noise affecting u(k) , denoted as w(k) in equation (2.1), we consider the correlation between the noise samples. In order to account for this correlation, we can use an AR model of order 20 derived in the previous section (i.e. the one used for the generation of the measurement noise on synthetic HR data). The AR model is driven by a stationary white noise ϵ(k) , with zero mean and an unknown variance $\sigma^{2}$ . Using the model and the equations for the driving noise, we can easily calculate $\Sigma_{w}$ as


(2.6) $$\Sigma_{w}=\sigma^{2}\;{\left(A^{T}A\right)}^{-1}.$$

Here, A represents a square *n*-dimensional lower triangular Toeplitz matrix. Its first column is given by $[1,a1,a2,a3,\ldots ,a20,0,\ldots ,0{]}^{T}$ .

Once defined $\Sigma_{u}$ and $\Sigma_{w}$ , equation (2.3) turns into


(2.7) $$\hat{u}={\left(A^{T}A+\gamma F^{T}F\right)}^{-1}A^{T}Ay.$$

Here, $\gamma =\sigma^{2}/\lambda^{2}$ is a regularization parameter that balances the fidelity to the data (first term in the cost function of equation (2.2)) and the smoothness of the estimate (second term in the cost function of equation (2.2)). Larger values of γ result in oversmoothing, where the estimate $\hat{u}$ is less influenced by the measurements y . On the other hand, smaller values of γ lead to undersmoothing, where the estimate $\hat{u}$ accurately fits the measurements y but does not effectively attenuate the noise.

However, the value of γ is unknown because both $\sigma^{2}$ and $\lambda^{2}$ are unknown. To estimate the value of γ , we use the maximum likelihood regularization criterion suggested by De Nicolao *et al*. [24]. By iteratively solving the problem of equation (2.6) for several trial values of γ , we look for the value of γ that satisfies the following condition:


(2.8) $$\frac{\text{WRSS}\left(\gamma \right)}{n-q\left(\gamma \right)}=\gamma \frac{\text{WESS}\left(\gamma \right)}{q\left(\gamma \right)},$$

where $\text{WRSS}\left(\gamma \right)={\left(y-\hat{u}\right)}^{T}A^{T}A{\left(y-\hat{u}\right)}^{T}$ is the weighted residual sum of squares, $\text{WESS}\left(\gamma \right)={\hat{u}}^{T}F^{T}F\hat{u}$ is the weighted estimates sum of squares and $q\left(\gamma \right)=\text{trace}\left[A^{T}{\left(A^{T}A+\gamma F^{T}F\right)}^{-1}A)\right]$ is the degrees of freedom of the estimator.

In this framework, one can also calculate the covariance of the estimation error $\tilde{u}=u-\hat{u}$ as follows:


(2.9) $$\text{cov}\left(\tilde{u}\right)=\sigma^{2}{\left(A^{T}A+\gamma F^{T}F\right)}^{-1}.$$

The square root of the diagonal elements of this covariance matrix can be used to determine the confidence interval (CI) of the estimate $\hat{u}$ .

#### 2.3.2. Tracking signal-to-noise ratio time-variability: windows segmentation and signal reconciliation

In order to optimize the computation time and capture the possible SNR time-variability in the HR data, it is advantageous to divide the signal into smaller windows. Indeed, this approach may allow to track the variation over time of the statistical characteristics of HR data, which may be caused by different activities performed throughout the day. The sample size for each window has been empirically set to 200 samples, striking a balance between computation speed and pattern detection in the data. This methodology also allows for the relaxation of the assumption of stationary measurement noise, which was introduced for the driving noise of the AR model. A similar approach was utilized by Camerlingo *et al*. [25] for the blood glucose concentration signal.

During the signal segmentation process, each window was created with 20 overlapping samples to ensure a smoother transition between consecutive windows during the subsequent smoothed signal reconciliation step.

The signal reconciliation step involves combining the filtered windows to obtain the fully processed signal. By concatenating the signals of each window without proper reconciliation, the resulting signal may exhibit spikes and abrupt changes that are not physiologically plausible and do not exist in the original signal. To address this issue, the overlapping sections of consecutive windows are utilized to connect the windows smoothly. This is achieved by applying a Gaussian kernel technique that assigns weights to the corresponding samples of the two overlapping sections, resulting in a seamless transition.

### 2.4. The two comparators: moving average and local regression filters

As a reference and baseline filtering methodology, we used two different state-of-the-art methodologies: moving average (MA) and local regression (LOESS) algorithms. The MA technique is a commonly used signal processing method that aims to reduce noise and smooth the signal by averaging neighbouring data points within a specified window and is widely used in the signal processing of biological signals [26–28]. In our implementation, we tested different MA window sizes, ranging from 5 to 35 samples. The local regression algorithm is based on weighted linear least squares and a second-degree polynomial model. The window size for this methodology has been fine tuned, resulting in a size of 80 samples.

### 2.5. Evaluation metrics

To assess the performance of BF and the comparators, we employed standard metrics such as the root mean square error (RMSE), mean absolute relative difference (MARD) and mean absolute error (MAE), all in respect to the ground truth, as evaluation metrics, which are defined, respectively, as


$$\text{RMSE}=\sqrt{\frac{1}{n}\sum_{i=1}^{n}(\hat{u}(i)-u(i){)}^{2}},$$

$$\text{MARD}=100\frac{1}{n}\sum_{i=1}^{n}\left|\frac{\hat{u}(i)-u(i)}{u(i)}\right|,$$

$$\text{MAE}=\frac{\sum_{i=1}^{n}\left|\hat{u}(i)-u(i)\right|}{n}.$$

To ensure that all outputs from the different methodologies align with the original data, we rounded the values to integers, similar to the output of the sensor itself.

## 3. Results

The following section presents an analysis of the achieved outcomes through the application of BF algorithm to HR data. First, we will present the evaluation of BF performance on the synthetic dataset, where the ability to correctly reconstruct the HR data can be quantified since the ground truth is available. On this dataset, we will also compare BF versus MA approaches. Then, we will assess BF ability of learning the statistical characteristics of HR time-series and smoothing them on real-life HR data (the second subset of the data) to also prove its practical applicability. The data and code used to recreate these results are available as electronic supplementary material.

### 3.1. Assessment of Bayesian filtering performance on synthetic heart-rate data

As anticipated, first the BF algorithm has been evaluated on the synthetic HR dataset, which allowed us to analyse the ability of the BF procedure to smooth the signal, retrieve the underlying ground truth HR, estimate the SNR and quantify the noise variance.

**Figure 3.**
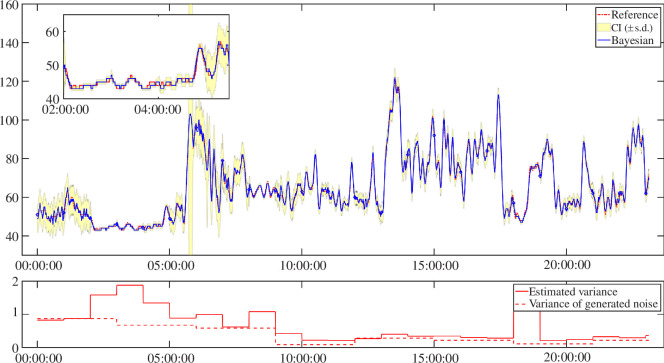
Resulting filtered signal obtained by the Bayesian methodology. In red, the ground truth signal used as reference. In blue, with confidence intervals (CI) as yellow area, the estimated smoothed signal. In the bottom plot, as a continuous red line, the estimated noise variance by the Bayesian smoothing algorithm, and the simulated noise variance in a thin dashed red line.

Figure 3 shows an example of the outcome that can be obtained from the application of the BF algorithm to HR data. The top panel depicts the ground truth HR data (red line), the smoothed HR signal obtained with the BF procedure (blue line) from synthetic noisy HR values (not shown here for sake of simplicity), and the confidence interval of the HR estimation (yellow band), defined as one standard deviation (s.d.). Comparing the two profiles, it can be appreciated how the smoothed HR signal is very close to the ground truth reference HR data, removing most of the noise while preserving the underlying variability of the ground truth HR signal. Moreover, by looking at the yellow band it can be seen that the reference signal is always inside the confidence interval, showing an optimal estimation precision.

The bottom panel of figure 3 shows, instead, the estimation of noise variance compared with the generated time varying profile used in the generation of the noisy benchmark signal. Overall, a visual inspection of the resulting plot shows a good estimation of the noise variance by the BF. With reference to the self-learning feature and the estimation of the SNR, we can observe that only in two instances does the BF algorithm overestimate the noise variance. The first instance is during nighttime sleep (i.e. from 02.00 to 05.00) where the overall variability of the signal is low, but the BF estimated high noise variability, possibly removing more information than the sole added noise, while still in strict adherence to the ground truth signal. The second instance is around 18.00, when we have a rapid increase in HR, probably due to the subject starting to move after a long resting phase. In this scenario, the BF algorithm fits a curve of the increase, considering all fluctuations as noise, again possibly removing some relevant information about the transient period.

From a quantitative point of view, the resulting smoothed signal achieved RMSE of 1.21 bpm, MAE of 0.85 bpm and MARD of 1.36%. Overall, we can conclude that BF algorithm performs well and that its adaptive and self-learning abilities have been proved.

**Figure 4.**
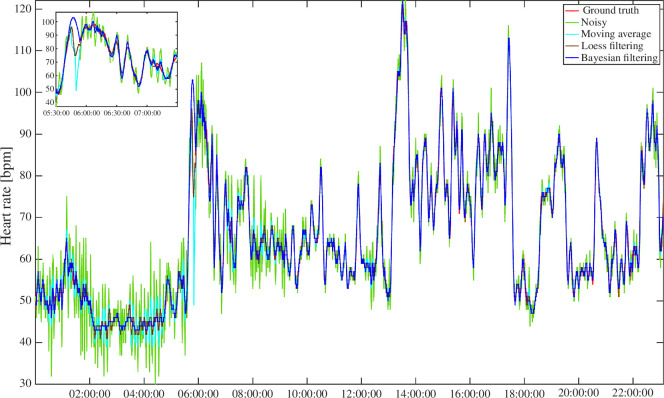
Comparison of input and resulting smoothed signals. The BF signal in blue is almost overlapping with the ground truth signal in red. The MA with 25 samples window signal (cyan) instead follows the overall shape of the noisy signal, in green, only reducing its amplitude in the night period, when the generated noise variance is higher. The LOESS signal performs similarly to the MA but has more filtering power.

Moving to the comparison with state-of-the-art MA filter, figure 4 reports the noisy synthetic HR data (green), the ground truth reference signal (red), the signal resulting from the MA with 25 samples window (cyan), the signal from the LOESS (brown) and the one estimated by the BF (blue).

From a visual inspection, we can observe that all methods allow smoothing the noisy HR data. However, both the MA and the LOESS methods perform suboptimally, e.g. undersmoothing in the first part of the time series, where the MA is still noisy and oscillating, and the LOESS while performing better than MA, still has oscillations. This is evident by considering numerical results of the evaluation of the global accuracy in reconstructing the ground truth HR profile. In fact, BF achieves lower values than both MA and LOESS for all considered metrics: RMSE of 1.21 bpm versus 1.83 bpm and 1.55 bpm, MAE of 0.85 bpm versus 1.32 bpm and 1.06 bpm and MARD of 1.35% versus 2.22% and 1.77% for BF, MA and LOESS, respectively.

The results of the comparison are important to highlight the differences, in terms of smoothing behaviour, between BF and MA methods. The MA closely tracks the noisy signal, although it provides only partial noise reduction. The LOESS improves the noisy signal especially during the daytime, but has issues during the nighttime, where it is not able to perform a satisfactory filtering. In contrast, the BF method exhibits a significantly different performance. It not only accurately follows the underlying signal’s trends and variations over time but also excels in noise elimination, resulting in a filtered HR signal that closely aligns with the ground truth HR signal. This is particularly evident during the nighttime (the RMSE for different day period is shown in table 1), during which both the MA and the LOESS are not able to remove most of the added noise, resulting in a signal that does not align with the ground truth. Table 1 also reports numeric results of all window sizes tested for the MA.

**Table 1.** Numeric results in terms of RMSE, MAE and MARD of the tested filtering techniques in relation to the extracted ground truth signal.

	overall			daytime			nighttime (00.00–06.00)		
	RMSE	MARD	MAE	RMSE	MARD	MAE	RMSE	MARD	MAE
BF	1.21	1.35	0.85	1.22	1.21	0.86	1.18	1.65	0.82
MA_5_	3.08	4.62	2.75	3.14	3.30	2.28	5.39	8.80	4.25
MA_15_	2.84	3.46	2.04	2.33	2.43	1.67	4.03	6.73	3.23
MA_25_	1.83	2.22	1.32	1.57	1.63	1.11	2.51	4.13	1.99
MA_35_	1.93	1.86	1.43	1.58	1.65	1.12	1.40	4.33	1.80
LOESS	1.55	1.77	1.06	1.30	1.31	1.93	2.1	3.31	1.66

**Figure 5.**
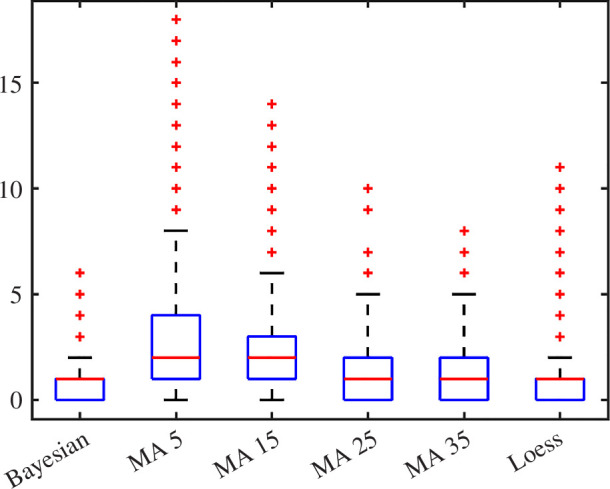
Box plot of residual distributions among all tested methods. The distribution has been tested significantly different against all comparators, thus confirming the improved performance of the BF in noise reduction and adherence to the ground truth signal.

To further elucidate the comparative performance of different methods, we conducted a paired *t*‐test to examine whether the disparities in residuals, shown in figure 5, between the MA with the tested window sizes and BF, and between the LOESS and BF methods were statistically significant. The results of the paired *t*‐test indicated that the differences were indeed statistically significant (*p* < 0.05) for all methodologies, underscoring the superior performance of the BF method in terms of residual reduction. This statistical validation confirms our earlier observations, emphasizing the enhanced noise reduction and signal fidelity achieved by BF when compared with other state-of-the-art methodologies.

### 3.2. Assessment on real-life heart-rate data

The validation of the BF algorithm was conducted on the second dataset, i.e. one day of HR data collected in free living conditions in individuals with amyotrophic lateral sclerosis or multiple sclerosis. It is important to evidence that this dataset can be considered as a sort of test set, these HR traces being distinct from those used in the previous section. This choice ensures that the performance of the BF algorithm remains sufficiently consistent when applied to previously unseen data, reinforcing its reliability and supporting the potential generalizability.

**Figure 6.**
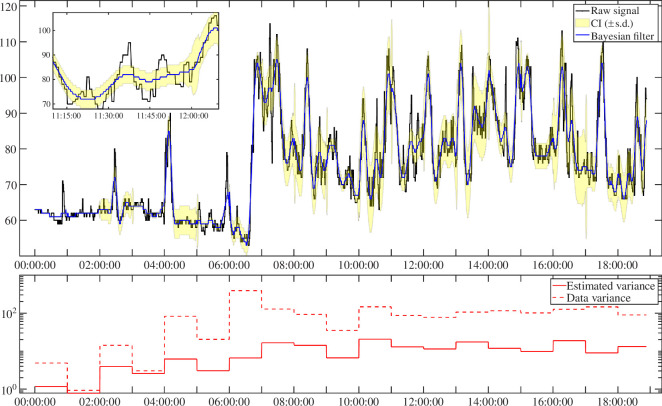
Sample data used as validation of the smoothing methodology. These data come from a different dataset from the training data to ensure the performance is maintained on never-seen data. The bottom panel shows the estimated and data variance in a logarithmic scale.

A representative signal from the validation dataset, as depicted in figure 6, showcases the capabilities of the BF algorithm in processing real-world HR data. Notably, the BF output signal aligns closely with the reference data, successfully mitigating sharp peaks, particularly during nighttime and periods of mild activity. An example is observed around 11.30, where the BF algorithm effectively eliminates two peaks, reducing data variability while preserving the overall heart-rate profile. The RMSE between the smoothed signal and the reference is measured at 1.29 bpm, surpassing the performance of the MA applied to the same validation signal (not shown in the figure), which yields an RMSE of 2.1 bpm. This capacity to preserve the HR profile while mitigating high-frequency fluctuations stands out as a distinctive and valuable characteristic of the BF technique.

The estimation of noise variance provided as outcome by the BF method could play an important role, possibly offering insights into the activity level of the subject. Moreover, when dealing with biological time-series corrupted by measurement noise, it is important to identify its variance in order, e.g. to understand if a specific portion of data is reliable/unreliable or if the clinician/user can no longer trust the value provided. The variance estimated by the BF also reflects the amount of signal the filter is able to remove, thus giving more information on the relative BF performance. Figure 6 shows in the bottom panel the estimated variance by the BF and the total variance calculated from the data in each window, in logarithmic scale. As expected, the variance estimated by the BF is always lower than the one of the data. The difference between the total variance of the data and the noise variance estimated by BF is smaller during nighttime. This is due to the fact that, at nighttime, physiological variations of HR are very limited and, correctly, BF attributes all the variance to the measurement noise. During daytime, the estimated noise variance by BF is much smaller than the total variance, and this is probably due to the activities performed during the day, which can change drastically the HR, while also introducing artifacts due to movement. Finally, we can also see that in the presence of physical activity, performed between 06.00 and 08.00, where there is high variability in the HR data, the BF method correctly attributes a very limited amount of the total data variance to noise. In conclusion, by capturing and discriminating the variations associated with activity and movement, the noise variance estimation might provide valuable information for further physiological analyses. Further research is needed to explore the possible connection between this estimation and daily events.

## 4. Conclusion

In this work, we set out to develop and evaluate a BF algorithm to improve the quality of HR data collected from wearable devices. The self-learning and adaptive algorithm has been applied to different datasets showing its effectiveness as a preprocessing technique for HR data analysis. Through comprehensive development, fine-tuning and rigorous evaluation, we have established the utility of BF in improving signal quality, reducing noise and preserving essential physiological information. Notably, the MAE result of the BF technique on the synthetic signal is lower than the MAE of consumer wearable HR monitoring smartwatches (e.g. 4.6 bpm for the Apple Watch 4 [29]). This shows how this technique improves the quality of the signal, tackling the noise of the device while preserving the properties of the signal without introducing a larger error than the measurement error by the device itself.

The successful application of BF to real-world data from a separate validation dataset underscores its effectiveness, supporting its potential generalizability. The ability to adapt to unseen data and maintain the fidelity of HR profiles while minimizing noise highlights the practicality of BF in various applications, including health monitoring and clinical research. Additionally, the estimation of noise variance provided by BF enriches the information of the HR signal, offering a valuable supplementary metric for physiological analysis. Future works will explore possible exploitation of extracted information from signals, such as possible correlations between estimated noise variance and physical activity or the clinical relevance of such information.

In conclusion, BF emerges as a promising tool for enhancing the accuracy and utility of vital signals collected via commercial wearable devices, e.g. HR data acquired with smartwatches, making these devices more reliable and more exploitable in clinical research and also clinical practice. Finally, its applicability to real-world scenarios, coupled with its noise reduction capabilities, positions it as a valuable asset in the field of physiological signal processing.

## Data Availability

The code and data to reproduce the figures in the paper are available at: [30]. Supplementary material is available online [31].
